# Self-adhesive flowable resin composite for occluso-proximal restorations in primary teeth: restorative time and fracture resistance analysis

**DOI:** 10.1590/1678-7765-2026-0045

**Published:** 2026-07-27

**Authors:** Tyler Zanella Brustolin, Cleber Paradzinski Cavalheiro, Duvan Cala Castillo, Gabriel Kalil Rocha Pereira, Tathiane Larissa Lenzi

**Affiliations:** 1 Universidade Federal do Rio Grande do Sul Faculdade de Odontologia Porto Alegre RS Brasil Universidade Federal do Rio Grande do Sul, Faculdade de Odontologia, Porto Alegre, RS, Brasil; 2 Universidade Federal do Rio Grande do Sul Programa de Pós-Graduação em Odontologia Faculdade de Odontologia Porto Alegre RS Brasil Universidade Federal do Rio Grande do Sul, Faculdade de Odontologia, Programa de Pós-Graduação em Odontologia, Porto Alegre, RS, Brasil; 3 Universidade Federal de Santa Maria Programa de Pós-Graduação em Ciências Odontológicas Santa Maria RS Brasil Universidade Federal de Santa Maria, Programa de Pós-Graduação em Ciências Odontológicas, Santa Maria, RS, Brasil

**Keywords:** Tooth, deciduous, Composite resins, *In vitro* techniques, Pediatric dentistry, Fracture resistance

## Abstract

Restoring occluso-proximal cavities in primary molars is clinically challenging due to their anatomical characteristics and the difficulty of matrix adaptation. Although resin composites are widely used, their technique sensitivity has led to simpler alternatives, such as self-adhesive flowable composites, whose performance in primary teeth remains unclear.

**Objective:**

This *in vitro* study evaluated the restorative time and fracture resistance of occluso-proximal restorations in primary molars using a self-adhesive flowable resin composite, a flowable resin composite, and a conventional resin composite.

**Methodology:**

Thirty primary molars received two standardized occluso-proximal cavities and were randomly assigned to three groups (n=10): (1) self-adhesive flowable resin composite (Yflow SA), with the entire cavity restored; (2) universal adhesive (Scotchbond Universal) in self-etch mode + flowable resin composite (Filtek Z350 XT Flow), with the entire cavity restored; and (3) universal adhesive in self-etch mode + conventional resin composite (Filtek Z350 XT), inserted incrementally. Restoration time was measured using a digital chronometer, and all teeth underwent 14 days of pH cycling before the fracture resistance test. Mean fracture resistance values were analyzed using one-way ANOVA and Tukey's test. The Kruskal–Wallis and Dwass–Steel–Critchlow–Fligner tests were used to compare the restoration time among groups. Failure modes were analyzed descriptively.

**Results:**

The self-adhesive flowable resin composite required the shortest restoration time, followed by the flowable resin composite and the conventional resin composite (p<0.001). Fracture resistance was significantly lower in the self-adhesive flowable resin composite group compared to the flowable and conventional resin composite groups (p<0.01). Adhesive failures predominated in the self-adhesive flowable resin composite group (45%), while mixed failures were more frequent in the other restorative approaches (70–75%).

**Conclusion:**

In a laboratory setting, the use of a self-adhesive flowable resin composite for the entire cavity compromised the fracture resistance of occluso-proximal restorations in primary molars, despite reducing operative time.

## Introduction

Restoring occluso-proximal cavities in primary molars has always been a challenge for clinicians due to their anatomical particularities. The contact area is broad,^1^ and the cervical enamel rods are oriented occlusally, ending abruptly at the cervix and gradually becoming thinner, as in permanent teeth.^2^ Consequently, proper placement of matrix systems is more difficult, increasing the risk of cervical gaps.^3,4^

Although resin composites have been recommended to restore occluso-proximal carious lesions that do not extend beyond the proximal line angles,^2^ their placement is technique-sensitive and time-consuming.^5,6^ This can be particularly challenging in pediatric dentistry, where limited patient cooperation and reduced clinical time may compromise the quality of restorative procedures. Additionally, bonding to primary dentin may be more challenging than to permanent dentin due to its lower mineral content and higher tubular density, which may affect adhesive performance.^7-9^

Self-adhesive flowable resin composites have been developed to simplify restorative procedures by eliminating the need for a separate adhesive application. Their bonding mechanism relies on functional acidic methacrylate monomers that interact with the smear layer and promote superficial demineralization of the underlying dental substrate, allowing limited infiltration and contributing to micromechanical retention and potential chemical interaction.^10^ In contrast, flowable and conventional resin composites require a prior adhesive step for substrate infiltration and bonding.

It has been suggested, based on low-certainty evidence, that self-adhesive flowable resin composite restorations demonstrate clinical performance comparable to that of conventional and bulk-fill resin composites in permanent teeth, including in occluso-proximal cavities.^11^ To the best of our knowledge, the mechanical performance of self-adhesive flowable resin composites for restoring occluso-proximal cavities in primary molars has not yet been investigated.

Therefore, this *in vitro* study aimed to compare the fracture resistance of occluso-proximal restorations in primary molars performed using self-adhesive and conventional flowable resin composites with that of a conventional resin composite applied using the incremental technique. Furthermore, the time required to perform each restorative approach was evaluated.

## Methodology

This laboratory-based study followed the Checklist for Reporting In-Vitro Studies (CRIS) guidelines^12^ and was conducted in accordance with the principles of the Declaration of Helsinki (World Medical Association).

### Sample Size Calculation

The sample size was calculated using a spreadsheet-based calculator (Sample Size Calculator, version 2.0; Wan Nor Arifin, 2017). The calculation was based on the mean difference (63 Newton) and standard deviation (48 Newton) of the fracture resistance of occluso-proximal cavities in primary teeth restored with a conventional resin composite using the incremental technique or with a flowable resin composite placed in the entire cavity.^13^ A significance level of 5%, a statistical power of 80%, and a dropout rate of 10% were adopted. The minimum sample size was determined to be 10 teeth and 20 cavities per group.

### Selection and tooth preparation

A sample of 30 sound primary molars (15 first and 15 second maxillary or mandibular molars) was obtained from a human teeth biobank after approval of the research protocol by the Institutional Ethics Committee (No. 4.573.690). Only teeth without visible fracture lines were included. Following extraction, the specimens were stored in a 0.5% chloramine solution for disinfection. Each tooth was then positioned 1 mm below the cementoenamel junction and stabilized in PVC rings using self-curing acrylic resin (JET Clássico, São Paulo, SP, Brazil) to standardize handling during the restorative procedures.^14^

### Cavity preparation

Cavity preparations were performed by two trained operators. Each tooth received two standardized occluso-proximal cavities on the mesio-occlusal and disto-occlusal surfaces. Preparations were conducted using a #2068 truncated-cone diamond bur (Fava, São Paulo, SP, Brazil) mounted in a high-speed handpiece (KaVo, Joinville, SC, Brazil) under continuous water cooling. The cavities were prepared with the following dimensions: 4 mm cervico-occlusal height, 4 mm bucco-lingual/palatal width, and 2 mm mesio-distal depth.^13^ The dimensions were verified using a digital caliper (Absolute Digimatic, Mitutoyo, Tokyo, Japan) to ensure standardization. The cavities had slightly convergent walls toward the occlusal surface, a flat floor, rounded internal line angles, and a rectangular proximal box.

### Randomization

The greatest bucco-lingual/palatal and mesio-distal dimensions of each tooth crown were measured using a digital caliper (Absolute Digimatic, Mitutoyo, Tokyo, Japan). The sum of these measurements was used to distribute the teeth across the groups, ensuring similar tooth size distribution and the allocation of five first primary molars and five second primary molars to each group.^15^ Randomization was performed by a researcher who was not involved in the laboratory procedures. The specimens were then randomly assigned to three experimental groups (n=10) using a computer-generated random sequence (Random.org—Randomness and Integrity Services Ltd., Dublin, Ireland), according to the restorative material used. Allocation concealment was maintained with the use of sequentially numbered opaque containers, preventing the operator from knowing the group assignment before the restorative procedures.

**Group 1:** Self-adhesive flowable resin composite (Yflow SA, Yller Biomateriais, Pelotas, RS, Brazil) used to restore the entire cavity and placed in two 2-mm increments.

**Group 2:** Universal adhesive (Scotchbond Universal, Solventum, St. Paul, MN, USA) applied in the self-etch mode, followed by a flowable resin composite (Filtek Z350 XT Flow; Solventum, St. Paul, MN, USA) used to restore the entire cavity and placed in two 2-mm increments.

**Group 3:** Universal adhesive (Scotchbond Universal, Solventum, St. Paul, MN, USA) applied in the self-etch mode, followed by a conventional resin composite (Filtek Z350 XT; Solventum, St. Paul, MN, USA) placed using the incremental technique.

### Restorative procedures

All restorations were performed by a single trained operator who was calibrated before the study to standardize the procedure. The materials used are described in Figure 1. A Tofflemire matrix retainer (TDV, Pomerode, SC, Brazil) and a metallic matrix band (Golgran, São Caetano do Sul, SP, Brazil) were applied to each tooth. The mesio-occlusal cavity was restored first, followed by the disto-occlusal cavity. Restorative procedures were performed according to the assigned group, following the manufacturer's instructions. Each tooth received two restorations (mesio-occlusal and disto-occlusal) using the same restorative material assigned to its experimental group. The resin composite increments were measured using a millimeter probe (Golgran, São Caetano, SP, Brazil) and light curing with a light-emitting diode curing unit (Radii-cal, SDI, Victoria, AUS), and an irradiance of 1200 mW/cm^2^ was verified using the built-in radiometer of the light curing unit before each session. Polishing was performed using rubber points (Astropol, Ivoclar Vivadent, Schaan, Liechtenstein) one day after restoration.

**Figure 1:**
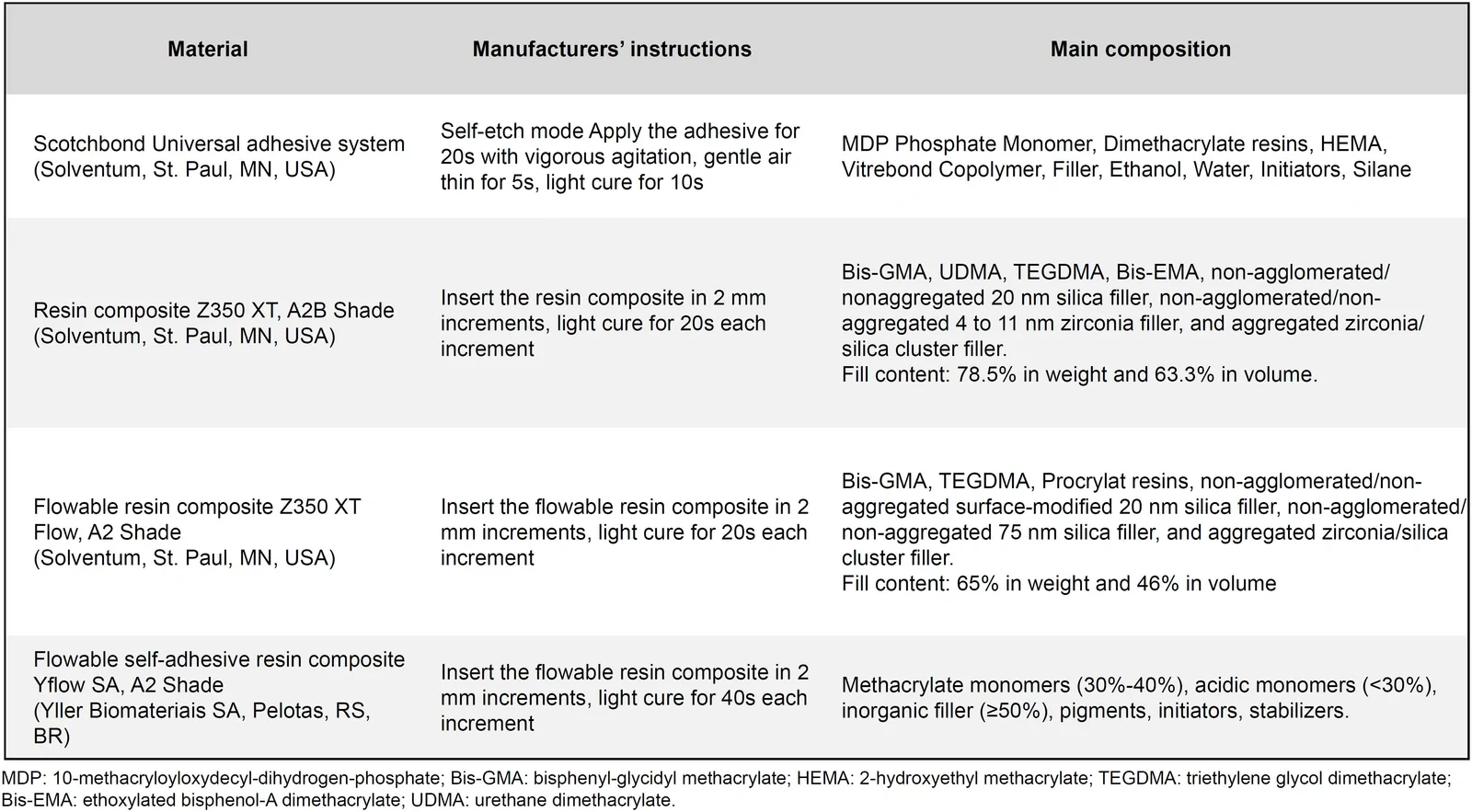
Main composition and manufacturers’ recommendations protocol of the materials used.

### Restorative time

The time required to perform each restoration was recorded in minutes by a trained assistant using a digital chronometer^16^ (Apple, Cupertino, CA, USA). The assistant was blinded to group allocation and only recorded the time required for each procedure. These measurements were obtained in a laboratory setting and do not account for clinical factors such as saliva control, behavior management, or matrix placement. For Groups 2 and 3, timing began with the application of the adhesive system to the mesio-occlusal cavity, whereas for Group 1, timing began with the placement of the first increment of the self-adhesive flowable resin composite in the first cavity (mesio-occlusal). In all groups, the chronometer was stopped after light-curing the final increment in the second cavity (disto-occlusal).

### Cariogenic challenge

All restored teeth were subjected to a cariogenic challenge by pH cycling before the fracture resistance test. The demineralizing solution contained 2.2 mM CaCl_2_, 2.2 mM NaH2PO4, and 50 mM acetic acid adjusted to pH 4.8, and the remineralizing solution contained 1.5 mM CaCl_2_, 0.9 mM NaH_2_PO_4_, and 0.15 M KCl adjusted to pH 7.0. Each tooth was individually immersed in 15mL of each solution for 8 h in the demineralizing solution and 16 h in the remineralizing solution. This procedure was performed for 14 days at room temperature without agitation, and the solutions were renewed daily.^17^

### Fracture resistance

Each restored tooth was numbered according to the randomization sequence to ensure blinding of the operator performing the mechanical testing. The teeth were individually mounted in a universal testing machine (EZ-SX series, Shimadzu Corp., Kyoto, Japan) and subjected to a compressive axial load applied to the center of each occluso-proximal restoration (mesio-occlusal first, followed by the disto-occlusal restoration), parallel to the long axis of the tooth, using a round-end steel device (6 mm in diameter) at a crosshead speed of 1 mm/min. The load was applied until fracture occurred, at which point the machine stopped automatically. The load required to fracture each specimen was expressed in Newton (N).

### Failure mode

After the fracture resistance test, a single trained examiner evaluated the failure mode of each occluso-proximal cavity under a stereomicroscope at 40x magnification. The failure mode was classified as follows: adhesive failure, when 100% of the bonded interface between the occluso-proximal cavity and the resin composite failed; cohesive failure, when 100% of the failure occurred within the resin composite; or mixed failure, when the failure was partially adhesive and partially cohesive.^18^

### Statistical analysis

The tooth was considered the experimental unit in this study. The fracture resistance values for each occluso-proximal restoration of the same tooth were averaged for statistical analysis. The mean fracture resistance for each experimental group was calculated as the average of the 10 teeth used in that group. Similarly, the time required to perform both the mesio-occlusal and disto-occlusal restorations (in minutes) was considered for statistical analysis. Thus, the mean restorative time for each experimental group was calculated based on the 10 teeth (20 restorations) included in that group.

The normality of data was assessed using the Shapiro–Wilk test, and homogeneity of variances was evaluated using Levene's test. Fracture resistance data were analyzed using one-way analysis of variance (ANOVA) and Tukey's *post hoc* tests. Restoration time was analyzed using the Kruskal–Wallis test and the Dwass–Steel–Critchlow–Fligner *post hoc* test. All statistical analyses were performed using Jamovi software (version 2.3; The Jamovi Project, Sydney, Australia), considering a significance level of 5%. Failure modes were analyzed descriptively.

## Results

The means and standard deviations for the time required to perform the restorations are presented in Table 1. Statistically significant differences were observed among all groups (p<0.001). The use of a self-adhesive flowable resin composite to restore the entire cavity resulted in the shortest restorative time (4.27±0.08 minutes), followed by the use of a flowable resin composite to restore the entire cavity (5.04±0.52 minutes). Conversely, the use of a conventional resin composite applied using the incremental technique led to the longest restorative time (11.58±0.52 minutes).

**Table 1:** Mean time required to perform the restorations (minutes), standard deviations, and 95% confidence intervals for all experimental groups.

Group	Restorative time	95% Confidence Interval
Group 1: Self-adhesive flowable resin entire cavity placed in two increments of 2 mm each	4.27 ± 0.08^A^	(4.21–4.33)
Group 2: Universal adhesive applied in the self-etch mode + Flowable resin composite entire cavity placed in two increments of 2 mm each.	5.04 ± 0.52^B^	(4.66–5.42)
Group 3: Universal adhesive applied in the self-etch mode + Conventional resin composite inserted by incremental technique.	11.58 ± 0.52^C^	(10.01–13.91)

* Different capital superscript letters indicate statistically significant differences between time required to perform the restorations (p < 0.001).

The mean fracture resistance values and standard deviations for all experimental groups are shown in Table 2. The use of a self-adhesive flowable resin composite to restore the entire cavity (728±183 N) significantly decreased the fracture resistance of occluso-proximal restorations in primary molars (p<0.01). However, no significant difference was observed between restorations using a flowable resin composite to restore the entire cavity (1,565±420 N) and those restored with a conventional resin composite (1,289±409 N) (p=0.207). The distribution of failure modes among the experimental groups is presented in Table 3. The self-adhesive flowable resin composite used to restore the entire cavity demonstrated the highest proportion of adhesive failures (45%). In contrast, the other restorative approaches showed a predominance of mixed failures (70–75%).

**Table 2:** The fracture resistance means (Newton), standard deviations, and 95% confidence intervals for all experimental groups.

Group	Fracture resistance	95% Confidence Interval
Group 1: Self-adhesive flowable resin entire cavity placed in two increments of 2 mm each	728 ± 183^B^	(597–859)
Group 2: Universal adhesive applied in the self-etch mode + Flowable resin composite entire cavity placed in two increments of 2 mm each	1,565 ± 420^A^	(1,265–1,865)
Group 3: Universal adhesive applied in the self-etch mode + Conventional resin composite inserted by incremental technique	1,289 ± 409A	(996–1,582)

* Different capital superscript letters indicate statistically significant differences between fracture resistance means among groups (p < 0.001).

**Table 3:** Distribution (%) of the failure modes for all experimental groups.

Group	Adhesive	Cohesive	Mixed
Group 1: Self-adhesive flowable resin entire cavity placed in two increments of 2 mm each	45%	25%	30%
Group 2: Universal adhesive applied in the self-etch mode + Flowable resin composite entire cavity placed in two increments of 2 mm each.	10%	15%	75%
Group 3: Universal adhesive applied in the self-etch mode + Conventional resin composite inserted by incremental technique.	15%	15%	70%

## Discussion

To our knowledge, this is the first study to investigate the fracture resistance and restorative time of occluso-proximal restorations in primary molars using a self-adhesive flowable resin composite. A flowable resin composite applied to the entire cavity and a conventional resin composite applied using the incremental technique were used as comparison protocols. The results demonstrated that the self-adhesive flowable resin composite required the shortest restorative time; however, it exhibited significantly lower fracture resistance values compared to the other restorative protocols.

The variability in children's cooperation during dental treatment^19^ further reinforces the need for restorative strategies that are simplified, less time-consuming, and easier to perform under challenging behavioral conditions. The use of flowable resin composites has been shown to reduce the time required to perform occluso-proximal restorations in primary teeth,^20^ making the procedure more manageable in pediatric patients. In this study, a self-adhesive flowable resin composite was evaluated as a simplified restorative alternative because it eliminates the need for a separate adhesive application step.

The use of a self-adhesive flowable resin composite required approximately 15% and 63% less time than the use of a flowable resin composite and a conventional resin composite, respectively. Both restorative materials were applied after using a mild universal adhesive in the self-etch mode.^21^ These findings indicate that eliminating the separate adhesive application step may reduce clinical chair time, which could be particularly advantageous in pediatric dentistry. However, while the time reduction observed compared with the conventional resin composite appears to be clinically meaningful, the approximately 15% reduction relative to the flowable resin composite protocol may have limited clinical relevance, as it represents only a small absolute decrease in operative time. Such a difference could easily be outweighed by other clinical variables, including patient cooperation and behavior management. Moreover, despite the gain in efficiency, the self-adhesive flowable resin composite did not provide comparable fracture resistance to the other restorative protocols.

A considerable number of studies have investigated the mechanical parameters of restorative materials by means of laboratory testing before their clinical application.^22^ Since extensive cavities reduce tooth resistance and mechanical stability is crucial for restoration longevity,^23,24^ evaluating fracture resistance is particularly relevant. This parameter reflects the load-bearing capacity and structural behavior of the tooth–restoration complex under functional conditions, rather than an intrinsic material property.

A systematic review and meta-analysis^25^ including five randomized clinical trials showed that self-adhesive flowable resin composites present clinical performance comparable to that of flowable resin composites in occlusal restorations of permanent teeth, with no significant differences in retention, secondary caries, marginal discoloration, or postoperative sensitivity. Additionally, low-certainty evidence suggests that the clinical performance of self-adhesive flowable resin composite restorations is comparable to that of conventional and bulk-fill resin composite restorations across different cavity configurations in permanent teeth.^11^

It is important to highlight that the self-adhesive flowable resin composite (Yflow SA; Yller Biomateriais, Pelotas, RS, Brazil) evaluated in this study was not included in the two aforementioned^11,25^ systematic reviews and meta-analyses. Therefore, the lower fracture resistance observed for this material may not be solely attributable to its self-adhesive nature but also to its overall formulation, including its viscosity and filler content. According to the manufacturer, Yflow SA contains approximately 50% inorganic filler, whereas the flowable resin composite (Filtek Z350 XT Flow; Solventum, St. Paul, MN, USA) contains 65% by weight and 46% by volume, and the conventional resin composite (Filtek Z350 XT; Solventum, St. Paul, MN, USA) contains 78.5% by weight and 63.3% by volume. It has been reported that the type and amount of filler, as well as the apparent viscosity, significantly influence the mechanical performance and wear resistance of flowable resin composites,^26^ which may explain the findings of this study. Lower apparent viscosity is commonly associated with a higher resin matrix content and reduced filler load, which may impair mechanical properties. Furthermore, low-viscosity materials may present greater intrinsic porosity and structural defects, which may contribute to reduced mechanical performance.^26^

Yflow SA was evaluated in this study because it was the only self-adhesive flowable resin composite commercially available on the Brazilian market during the experimental period. Unlike other commercial brands already established in the literature and evaluated in systematic reviews^11,25^ (e.g., Vertise Flow; Kerr and Beautifil Kids SA; Shofu), Yflow SA is a relatively new material for which scientific evidence remains limited, particularly regarding its fracture resistance.

The material formulation alone does not fully explain the observed outcomes; the adhesive interface also appears to have influenced the results. The higher incidence of adhesive failures (45%) observed with the self-adhesive flowable resin composite suggests that the adhesive interface may represent the critical factor affecting the mechanical performance of these restorations. This aligns with the lower fracture resistance observed for this group, indicating that the absence of a separate adhesive system may have compromised interfacial integrity. In fact, recent laboratory findings^27^ have shown that self-adhesive flowable resin composites, including Yflow SA, exhibit substantially lower microtensile bond strength (μTBS) to dentin than a flowable resin composite used after adhesive application. Such lower μTBS values support the notion that the bonding effectiveness of these materials may still represent a limiting factor. This behavior may be associated with their limited ability to effectively demineralize and infiltrate the dentin substrate, resulting in a less defined hybrid layer and weaker micromechanical interlocking.^10^ Such a mechanism may help explain the predominance of adhesive failures and the lower fracture resistance observed in this study. In contrast, the predominance of mixed failures (70–75%) in the flowable and conventional resin composite groups may reflect a more balanced stress distribution between the adhesive interface and the restorative material, which is consistent with the superior mechanical performance observed for these protocols.

This *in vitro* study presents some limitations. The restorations were performed without adjacent teeth, as each tooth was individually mounted and stabilized in PVC rings with self-curing acrylic resin to standardize handling. Additionally, the assessment of restorative time considered only the time required to perform the restorative procedures under controlled laboratory conditions. In clinical practice, operative time may be influenced by several additional factors, including patient cooperation and behavior management, quality of isolation, cavity accessibility, and operator-related variables, which were not accounted for in this study. Furthermore, the mechanical test used an axial compressive load applied parallel to the long axis of each occluso-proximal restoration. Clinically, lateral forces, as well as wear and fatigue effects, play an important role in the performance of occluso-proximal restorations, and achieving an appropriate proximal contact is essential to prevent food impaction and patient discomfort.^28,29^ It has been shown that thermomechanical cycling used to induce material fatigue does not significantly affect the fracture resistance of flowable resin composites in occluso-proximal restorations.^30^ In this sense, all restorations were subjected to a cariogenic challenge before fracture resistance testing to simulate oral environmental conditions that may impact restorative performance.^17,31^ Further studies assessing the fracture resistance of different self-adhesive flowable resin composites are needed to verify the consistency of these findings across materials with different compositions and properties.

## Conclusion

Based on this laboratory study, the use of self-adhesive flowable resin composite to restore the entire cavity compromises the fracture resistance of occluso-proximal restorations in primary molars, despite reducing operative time. In contrast, the flowable resin composite used with an adhesive system and the conventional resin composite exhibited comparable fracture resistance values.

## Data Availability

All data generated or analyzed during this study are included within the article.
